# Quantitative Comparison of HSF1 Activators

**DOI:** 10.1007/s12033-022-00467-3

**Published:** 2022-02-26

**Authors:** Christoph Steurer, Sarah Kerschbaum, Christina Wegrostek, Stefan Gabriel, Ali Hallaj, Viktoria Ortner, Thomas Czerny, Elisabeth Riegel

**Affiliations:** Department of Applied Life Sciences, University of Applied Sciences, FH Campus Wien, Helmut-Qualtinger-Gasse 2, 1030 Vienna, Austria

**Keywords:** Heat shock response, Neurodegenerative disease, HSF1, Luciferase assay

## Abstract

The heat shock response (HSR) pathway is a highly conserved rescue mechanism, which protects the cells from harmful insults disturbing the cellular protein homeostasis via expression of chaperones. Furthermore, it was demonstrated to play crucial roles in various diseases like neurodegeneration and cancer. For neurodegenerative diseases, an overexpression of chaperones is a potential therapeutic approach to clear the cells from non-functional protein aggregates. Therefore, activators of the HSR pathway and its master regulator HSF1 are under close observation. There are numerous HSR activators published in the literature using different model systems, experimental designs, and readout assays. The aim of this work was to provide a quantitative comparison of a broad range of published activators using a newly developed HSF responsive dual-luciferase cell line. Contrary to natural target genes, which are regulated by multiple input pathways, the artificial reporter exclusively reacts to HSF activity. In addition, the results were compared to endogenous heat shock protein expression. As a result, great differences in the intensity of pathway activation were observed. In addition, a parallel viability assessment revealed high variability in the specificity of the drugs. Furthermore, the differences seen compared to published data indicate that some activators exhibit tissue-specific differences leading to interesting assumptions about the regulation of HSF1.

**Supplementary Information:**

The online version contains supplementary material available at 10.1007/s12033-022-00467-3.

## Introduction

When Ritossa first discovered the heat shock response (HSR) in 1962, a whole new research field was initiated [1]. A vast amount of papers on this topic were published in the following years, until today. The heat shock response pathway has become more and more important, not only as a highly conserved cellular rescue mechanism after stressful insults and its correlation to thermal tolerance [2], but also because it plays an important role in wide-ranged varieties of diseases in humans, for instance neurodegenerative diseases and cancer [3–9].

The HSR can be triggered not only by heat, but by various stressors like changes in pH or oxygen levels, heavy metals, bacterial or viral infections, and various small molecules [10, 11]. In mammals, the central element in the activation of the HSR is the transcription factor Heat Shock Factor 1 (HSF1), which is a main trigger for the stress-regulated expression of members of the major heat shock protein (Hsp) families (e.g., Hsp72, Hsp25, Hsp40, Hsp60, Hsp100) [12]. These Hsps are well-explored chaperones, responsible for the correct folding of newly synthesized proteins as well as the re-folding of denatured proteins and, therefore, prevent aggregation of misfolded proteins and non-functional structures [13, 14]. Hsps are highly abundant. Of the more than 100 Hsps in the cell, Hsp72 (encoded by the HSPA1A and A1B genes) is highest upregulated after HSR activation [15, 16]. The promoter integrates different signaling pathways [17], but the main effect is mediated by the HSR and HSF1 activation. After a stressful insult (e.g., heat), HSF1 monomers trimerize, translocate into the nucleus, and bind to heat shock elements (HSEs) in the promoter regions of Hsps. In addition, HSF1 also undergoes several post-translational modifications (phosphorylation, acetylation, and sumoylation) during the activation process [18]. In the past, these modifications were considered essential for the activation, although a more recent publication suggests only minor fine-tuning potential for the phosphorylation [19].

Neurodegenerative diseases (e.g., Alzheimer’s disease, Huntington’s disease, Parkinson’s disease) are widely spread among the aging human population, and until today, the treatment options available only alleviate few symptoms, but no cure or even slowdown of the underlying molecular and organic malfunctions [20], although it is clear that aggregates of misfolded proteins (e.g., amyloid β, huntingtin, α-synuclein) are mostly responsible for these diseases. Activation of HSF1, as master regulator of the HSR, could provide help via increased chaperone production [3, 21–23]. Amyloids, plaques, inclusion bodies, and stress granules are all protein aggregates containing different proteins and can cause mild to severe damage to the cell. It was suggested that certain Hsps have the potential to clear the cell from specific protein aggregates, which can cause neurodegenerative diseases, like Alzheimer’s disease, Amyotrophic Lateral Sclerosis (ALS), and Parkinson’s disease [7, 24–26]. Recently, a neuroprotective mechanism, independent of chaperone expression, was also described for HSF1 [27, 28]. Apart from that, HSF1 has been shown to play a role in protection against oxidative stress [29] and is not only connected with proteotoxic but also metabolic stress [30]. This indicates that there are many more connections to other diseases yet to be found, where an activation of HSF1 might lead to promising therapeutic approaches. Cancer can also be linked to the HSR and its key players. In contrast to neurodegenerative diseases, where an over-activation of HSF1 is a promising approach to protect affected cells from further damage, cancer cells can use the protective function of the active HSF1 for promoting a malignant state [5, 6, 31].

In order to use HSF1 activation as a treatment for neurodegenerative diseases, the transcription factor has to be activated very specifically, without causing further cell stress or damage. A considerable number of molecules have been found with different activation potential and mechanisms. Geldanamycin, for instance, has shown to be a potent inhibitor of Hsp90 and, as a consequence, is a highly potent activator of HSF1 [32]. While geldanamycin itself was not successful in clinical trials due to high toxicity and limited bioavailability [33, 34], derivatives and analogs as well as other Hsp90 inhibitors such as SNX-2112 and its bioavailable pro-drug SNX-5422, showed promising results in mouse model studies [35] and was tested in phase I clinical trials for solid tumor cancers and lymphomas (Clinical Trials Identifier NCT00644072), for resistant lung adenocarcinoma (NCT01851096), neuroendocrine tumors (NCT02063958), and melanomas [36]. Co-inducers of the HSR, like bimoclomol, and its derivative arimoclomol, were found to be very effective in enhancing the heat shock response during already stressful conditions [37] and displayed promising results in clinical trials [38]. Other ongoing and upcoming trials with arimoclomol show a wide range of applications in various diseases, for instance, Niemann Pick Disease Type C (NCT02612129), Gaucher disease Type 1 or 3 (NCT03746587), Inclusion Body Myositis (NCT02753530), or ALS (NCT03836716, NCT03491462).

In this work, we established a stable luciferase reporter cell line to perform quantitative comparisons between potential HSF1 activators. Although much information on the activators can be gathered from the literature, a direct quantitative comparison is yet missing for most of the tested inducers. The aim was, therefore, to create a large set of directly comparable data that not only consider the potency of the activators tested, but also their effects on cell viability. The well-known inducers like H_2_O_2_, heavy metals, alcohols, pH, as well as heat were tested additionally to potential HSR-related drugs, which are currently under close observation for treatments in various diseases. To attach further importance to the gained results and correlate it to the endogenous target gene expression, we also determined Hsp activation in different cell types.

## Material and Methods

### Cell Lines and Culture Conditions

**Fig. 1:**
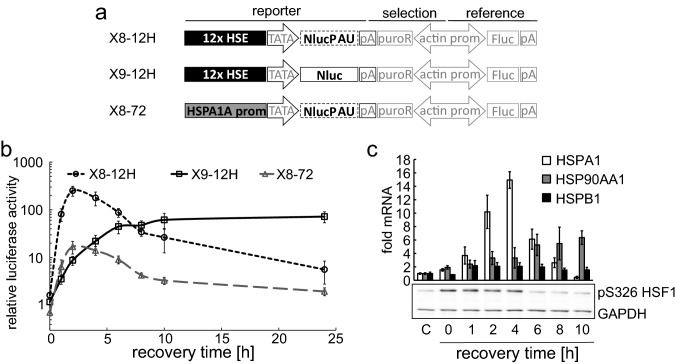
Luciferase reporter cell lines to detect HSF1 activation. A schematic presentation of the stably integrated dual-luciferase reporter constructs is shown in (**a**) (*HSE* heat shock element, *TATA* minimal promoter, *NlucPAU* Nluc with protein, and mRNA destabilizing sequences, *pA* polyadenylation signal, *puroR* puromycin resistance, *prom* promoter, *Fluc* firefly luciferase). The three different HSF1 reporter cell lines containing either 12 idealized HSEs in combination with Nluc (X8-12H) or NlucPAU (X9-12H) or the HSPA1A promoter in combination with NlucPAU (X8-72) were analyzed after heat treatment (10 min at 43 °C) for up to 24 h with luciferase measurement (**b**). *Y*-axis shows luciferase activity relative to control cells kept at 37 °C. *X*-axis shows recovery time after heat treatment. For correlation of the reporter signal to endogenous HSR pathway activation, relative HSPA1, HSP90AA1, and HSPB1 mRNA (**c**, upper panel) were determined with qPCR in X8-12H cells after heat treatment (10 min 43 °C) and active HSF1 was detected via phospho-specific antibody (pS326) with Western blot (**c**, lower panel) in HEK293 cells under the same conditions. For qPCR, all values were normalized to GAPDH mRNA expression and compared to control cells kept at 37 °C. All values are the means of at least three independent experiments, and error bars indicate standard error of the mean (SEM)

All cells were cultured in DMEM 4.5 g/L glucose (Pan Bio) with 10% fetal bovine serum (Pan Bio) and Penicillin/Streptomycin (HyClone) in a humidified environment at 37 °C and 5% CO_2_. T-REx™-293 cells (referred to as HEK293 cells further on) were purchased from Invitrogen, HeLa cells were purchased from CLS and immortalized WI38 (WI38 VA-13 subline 2RA) were purchased from ATCC. X8-72 (single clone #1), X8-12H (single clone #2), and X9-12H (single clone #13) were generated by introducing pGVL8 (for X8 cell lines, Fig. 1a) [39] or pGVL9 (for X9 cell line, Fig. 1a) backbones containing either the HSPA1A promoter or 12 × repeated HSE sites [40] into HEK293 cells with the PiggyBac transposon system [41]. For pGVL9, the reporter luciferase NlucPAU was changed to a non-degrading version of NanoLuc luciferase [42]. Due to poor transfection efficiency of fibroblast cell lines, the WI-rep2-12H (single clone #5) cells were generated with lentiviral transduction of immortalized WI38 cells with pLVrep2 12HSE where the pGVL9 12HSE dual luc + puromycin resistance cassette (see Fig. 1a) was integrated into a pHAGE lentiviral backbone (Addgene plasmid #50,919) [43] with NEBuilder cloning. Lentiviral particles were generated with Lenti-X Packaging single shots (Clonetech) in HEK293T cells.

### Heat Treatment and Induction

48 h after seeding, cells were incubated with different concentrations of established heat shock inducers diluted in DMEM with 10% FCS (DMEM complete) for 24 h. Stock solutions were prepared in recommended solvents and stored at − 20 °C: CdSO_4_ (CAS 7790-84-3, Carl Roth), AsNaO_2_ (CAS 7784-46-5, Sigma Aldrich), VER155008 (CAS 1134156-31-2, Santa Cruz), geldanamycin (CAS 30562-34-6, Carl Roth), SNX-2112 (CAS 908112-43-6, Cayman chemical), BGP-15 (CAS 66611-37-8, kindly provided by Tim Crul and László Vígh), arimoclomol (CAS 289893-25-0, Carbosynth), sulindac (CAS 38194-50-2, Santa Cruz), acetyl salicylic acid (CAS 50-78-2, Sigma Aldrich), mefenamic acid (CAS 61-68-7, Santa Cruz), ibuprofen (CAS 1189866-35-0, Santa Cruz), NaCl (CAS 7647-14-5, Carl Roth), sorbit (CAS 50-70-4, Carl Roth), carbenoxolone (CAS 7421-40-1, Sigma Aldrich), tosyl-L-phenylalanin-chlormethylketon (TPCK) (CAS 407-71-1, Applichem), MG132 (CAS 133407-82-6, Sigma Aldrich), celastrol (CAS 34157-83-0, Santa Cruz), resveratrol (CAS 501-36-0, Carl Roth), geranylgeranylacetone (CAS 6809-52-5, Sigma Aldrich), H_2_O_2_ (CAS 7722-84-1, Carl Roth), guanidinium (CAS 50-01-1, Carl Roth), and Tween20 (CAS 9005-64-5, Carl Roth). For pH titration, unbuffered DMEM (Sigma Aldrich, #D5030) was supplemented with 44 mM MES (CAS 1266615-59-1, Sigma Aldrich) (pH 4–7.4) or 44 mM Tris (CAS 77-86-1, Carl Roth) (pH 7.4–11) and the pH adjusted accordingly. pH, methanol (MetOH) (CAS 67-56-1, Carl Roth), and ethanol (EtOH) (CAS 64-17-5, Carl Roth) treatments were performed for 1 h and analyzed after 24 h recovery in DMEM pH 7.4.

Heat treatment was performed in 96-well plates in the Arktik™ Thermal Cycler (Thermo). Optimal heat transfer was ensured by placing the cell culture plates on an aluminum plate with wet filter paper and replacing the lid with a thin plastic foil. Cycling program was 5 min at 37 °C, followed by a preheating period of 2 min 30 s at the desired HS temperature + 5 °C in order to achieve faster temperature equilibration, 10 min at HS temperature, and 5 min cool down at 30 °C. The lid temperature was set to HS + 5 °C. For the recovery period, cells were placed in the incubator at 37 °C, 5% CO_2_.

For co-inducer experiments, cells were incubated with different inducer concentrations for 1 h before a 1 h heat treatment at 42 °C in a cell culture incubator was performed. Afterwards, cells were recovered at 37 °C with 5% CO_2_.

### Flow Cytometry

For dye exclusion assay, cells from one well of a 6-well plate were trypsinized, inactivated, and resuspended in 500 µL PBS with 10 µg/mL propidium iodide. The number of viable cells (from 10,000 total events) was analyzed with CytoFlex flow cytometer (Beckman Coulter).

### Luciferase Reporter Assay

The dual-luciferase experiments were performed with a Luminoskan Ascent (Thermo Scientific) [44]. For single-luciferase measurements, the Nluc substrate (6.25 mM Tris pH 7.5 and 3 µM coelenterazine) was dispensed and followed by immediate measurement and dispensation of stop solution.

### qPCR

Cell lysis, RNA isolation, cDNA synthesis, and qPCR reaction were described before [42]. The qPCRs for HSPA1 (detecting HSPA1A and HSPA1B coding for the Hsp72) and GAPDH were TaqMan assays, for HSP90AA (coding for Hsp90) and HSPB1 (coding for Hsp27) that were performed as SybrGreen assays. The oligos for HSPA1 were AACCAGGTGGCGCTGAAC (forward primer [fw]), TGGAAAGGCCAGTGCTTCAT (reverse primer [rv]), and AACACCGTGTTTGACGCGAAGCG (probe). For GAPDH, the oligos were GGAAGGTGAAGGTCGGAGTCAA (fw), ACCAGAGTTAAAAGCAGCCCTG, (rv) and ATTTGGTCGTATTGGGCGCCTGGTC (probe). The primers for HSP90AA1 were GAAGATGACCCTACTGCTGATGATACCAG (fw) and CGTTACCCCAATCTGTGAAAATAAACCAAC (rv). And the primers for HSPB1 were GCGTGTCCCTGGATGTCAACCACTT (fw) and ACTTGGCGGCAGTCTCATCGGA (rv).

### Viability with Resazurin

The resazurin assay was performed as described in [44].

### Western Blot

Whole cell protein extract preparation and western blot were performed as described previously [45]. For phospho-specific detection of HSF1, α-pS326 HSF1 (Abcam, ab76076; dilution 1:2000) and α-GAPDH (Santa Cruz, sc-25778; dilution 1:5000) were used as primary antibodies. Primary antibodies for detection of the heat shock proteins were Hsp72 (product of the HSPA1A gene), α-Hsp70 (Santa Cruz, sc-1060-R; dilution 1:5000), Hsp90 (product of HSP90AA1 gene), α-Hsp90 (Santa Cruz, sc-7947; dilution 1:1000), and Hsp27 (product of HSPB1 gene), α-Hsp27 (Cell Signal Technology, 2402S; dilution 1:1000). α-GAPDH (Santa Cruz, sc-25778; dilution 1:1000) was used as reference. As secondary antibody, goat anti-rabbit IgG-HRP conjugated (Santa Cruz, sc-2004) and mouse-IgGκ-binding protein HRP (Santa Cruz, sc-516102) were used in a 1:5000 dilution.

## Results

### HSF1 Reporter Cell Lines

Luciferase reporters utilizing Hsp promoter sequences have repeatedly been used for the detection of HSR activation. Here, we used an artificial promoter exclusively reacting to HSF activity, thus, providing high specificity by excluding any other transcription factors or signaling pathways taking part in stress-induced Hsp expression. We previously used such cell lines for analyzing kinetics of different heat treatments [40] and heavy metal treatments [42]. For this work, we created a novel, optimized HSF reporter cell line (Fig. 1a). We used 12 × multimerized consensus HSEs [46] upstream of an artificial minimal promoter and two distinct versions of NanoLuc [47] as reporter gene. To achieve high signal intensity, the very stable wild-type NanoLuc (Nluc) was used (X9-12H). For kinetic analyses, an Nluc with a very short half-life (NlucPAU) was integrated into the reporter construct (X8-12H) [42]. Furthermore, a cell line containing an HSPA1A promoter [40] in combination with NlucPAU (X8-72) was generated. All three cell lines were tested under heat stress conditions (10 min 43 °C) and analyzed up to 24 h post-heat stress (Fig. 1b). For the reporter cell lines with the non-stable NlucPAU (X8-12H and X8-72), peak levels of activation were reached early, after 2–4 h, whereas the reporter cell line with the reporter gene with a longer protein half-life (Nluc) accumulated further, even when HSF1 was no longer active and reached maximum activation at about 10 h post-heat treatment and the luciferase level stayed high until 24 h post treatment. At this late time point, there was almost no signal left in the other two cell lines. In consistency with our previous study [40], a direct comparison of the HSPA1A promoter to the artificial HSE promoter showed higher induction levels for the multimerized HSE reporter (17-fold compared to 250-fold after 2 h recovery time). However, the kinetics for the HSPA1A reporter and the HSE reporter were in concordance.

To further confirm the correlation between the artificial HSF1 luciferase reporter, the actual activity of HSF1, and the endogenous HSR pathway, we also examined the endogenous HSF1 target genes HSPA1 (Hsp72), HSPB1 (Hsp27), and HSP90AA1 (Hsp90) on the level of mRNA (Fig. 1c, upper panel) as well as HSPA1 (Hsp72) on protein level (Fig.S1). In addition, we examined the activation level of HSF1 with a pS326-specific antibody (Fig. 1c, lower panel). Our observations indicate that under these conditions, HSF1 is activated up to 4 h post-heat treatment, after which HSF1 phosphorylation on S326 is abolished. The target genes HSPA1 and HSPB1 also reach maximal expression after 4 h with 14.9- and 2.1-fold induction, respectively. The expression of HSP90AA1 is further increasing and reaches 6.4-fold activation after 10 h post-heat treatment (Fig. 1c).

On the protein level, the HEK293 cells generally showed low activation of the target gene HSPA1 (Hsp72) because of high basal expression levels [16]. Therefore, we also looked at two other cell lines (HeLa and the human fibroblast cell line WI38) and saw that Hsp72 protein upregulation is prolonged compared to mRNA, indicating a slower turnover of proteins compared to mRNA (Fig. S1).

In conclusion, the activation kinetics of the endogenous target protein Hsp72 was similar to the results gained with the artificial HSE-containing promotor in the Nluc reporter cell lines. Additionally, the kinetics of HSPA1 and HSPB1 mRNA expression and HSF1 phosphorylation demonstrated that the artificial HSE reporter, with the short-lived reporter protein NlucPAU, accurately represents the kinetics of HSF1 activity.

### Induction Kinetics and Cell Viability

Compared to heat stress, where the stressful effect to the cell is typically short, other stressors or chemical inducers might show different kinetics of HSF1 activation. Even short pulses of heat treatment of the X9-12H cell line, which contain the accumulating luciferase, resulted in strong signals after 24 h, although HSF1 activation itself was long attenuated at this time point. We, therefore, proposed that this reporter cell line is best suited to test HSR inducers with unknown activation kinetics for their ability to specifically activate HSF1.

**Fig. 2:**
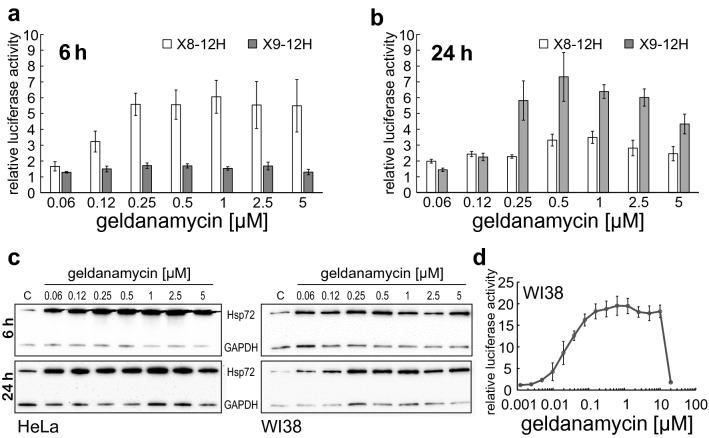
HSF1 activation with geldanamycin. X8-12H and X9-12H (**a** and **b**) or WI38-rep2-12H (**d**) cells were incubated with increasing concentrations of geldanamycin and Nluc activity was measured after 6 h (**a**) or 24 h (**b** and **d**) incubation time. *Y*-axis shows relative luciferase activity as Nluc signal of treated cells divided by untreated control cells. For Western blot analysis, (**c**) HeLa (left) and WI38 (right) cells were incubated with different concentrations of geldanamycin and whole cell protein extracts were taken after 6 h (upper panel) or 24 h (lower panel). Control cells were untreated. Western blot was performed with primary antibodies against Hsp72 and GAPDH. For luciferase experiments, all values show means of at least three independent experiments, and error bars indicate SEM

In order to compare the suitability of the two different reporter cell lines X8-12H and X9-12H, we used geldanamycin as a model substance. Reporter activation was analyzed after 6 (Fig. 2a) and 24 h (Fig. 2b). While the X8-12H reporter already showed activation at 0.06 µM and reached its maximum and plateau level at 0.25 µM the 6 h point of measure, the X9-12H reporter never reached levels above twofold. After 24 h, activation in the X8-12H cell line was reduced but still detectable at geldanamycin concentrations starting at 0.12 µM, but lower compared to 6 h. In contrast, activation of the X9-12H cell line started at 0.12 µM with peak inductions at 0.25 µM to 5 µM after 24 h. For both cell lines, there was a decrease from 1 to 5 µM, probably due to the toxic effects that geldanamycin has at these concentrations after 24 h incubation.

To clarify the kinetics of the geldanamycin-mediated HSR not only in respect to HSF1 activation but also regarding the activation of endogenous target genes, we looked at endogenous Hsp levels (Figs. 2c and S2). In contrast to the HSF1 reporter data, the Western blot for Hsp72 showed already high induction levels at the lowest geldanamycin concentration tested (0.06 µM) and with similar expression patterns for 6 and 24 h (Fig. 2c). For Hsp27, on the other hand, the expression pattern correlates well with the reporter data, whereas Hsp90 was not induced at all at the tested conditions (Fig. S2). Although there are differences between the expression patterns of different endogenous Hsps and the HSF1 reporter activity, we conclude that results from a 24 h incubation of the X9-12H cell line with geldanamycin well capture HSF1 activity integrated over a longer period of time. The reporter cell line X8-12H on the other hand is suitable for kinetic measurements, in particular for short-time applications of stressors.

In order to determine if the more sensitive response seen on Hsp72 protein level compared to the HEK293-based reporter cell lines is caused by an HSF1 independent mechanism, or due to the sensitivity of the cell lines used, we also looked at a WI38-based reporter cell line (Fig. 2d). In this case, reporter activity started at about 10 times lower concentrations (0.005 µM) compared to the X9-12H cell line. We, therefore, concluded that the differences seen for geldanamycin activation of Hsp72 result from different sensitivities in different cell lines. To further prove that the measurement after 24 h is also suitable for different inducers we analyzed the kinetics of the drugs carbenoxolone, TPCK and MG132 (Fig. S3a) as well as the response to heavy metals (cadmium and arsenic) and low pH (Fig. S3b). Although the onset and intensity of reporter activation varied between the inducers, all of them showed peak activation after 24 h.

**Fig. 3:**
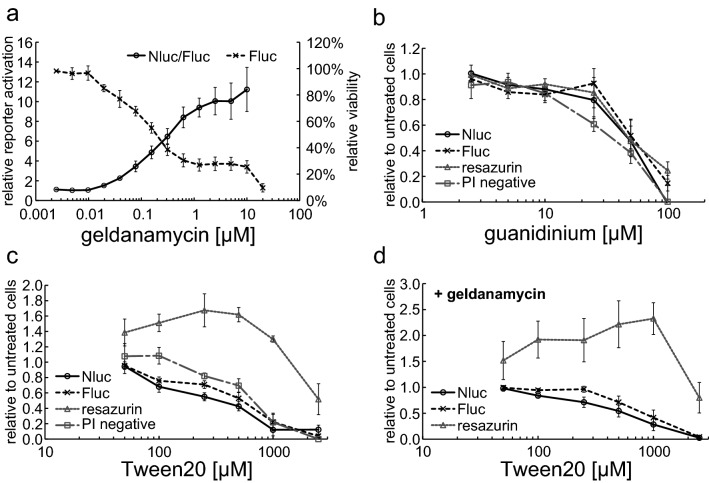
Dual-luciferase assay for viability assessment. For dual-luciferase measurement, (**a**) X9-12H cells were treated with different concentrations of geldanamycin for 24 h. Left *Y*-axis shows relative luciferase activity as Nluc/Fluc signal compared to untreated control cells. Right *y*-axis shows relative viability as Fluc signal relative to untreated control cells. For comparison of different viability parameters (**b**–**d**) X9-12H cells were treated with increasing concentrations of guanidinium (**b**) and Tween20 (**c**) or 2.5 µM geldanamycin combined with different concentrations of Tween20 (**d**) for 6 h. *Y*-axis shows signal (Nluc, Fluc, resazurin) or cell count (PI negative) relative to cells without guanidinium or Tween20. Nluc and Fluc were measured with a dual-luciferase assay, metabolic activity with a resazurin assay (fluorescence), and PI-negative cells were counted with a flow cytometer. All values show the mean of at least three independent experiments, error bars indicate SEM, Student’s *t* test was applied to estimate significance (see text)

A critical point to differentiate between a general stress response and a specific activation of HSF1 is to assess cell viability in parallel to the reporter data. Monitoring of the cell metabolism can serve as an indirect parameter for cell viability, using, for example, a resazurin assay. For special applications, where viability is determined after a short incubation time or when substances that are suspected to have an influence on cell metabolism rather than cell viability, an assay based on the metabolic activity of the cells might not be accurate enough. To overcome this problem, we included a second, constitutively expressed luciferase (Fluc) as an internal reference in our HSF1 reporter cell line. When looking at HSF1 activation with geldanamycin, using the dual-luciferase assay, we saw that the cell viability determined by Fluc measurement started to decrease at concentrations as low as 0.02 µM geldanamycin (Fig. 3a, Fluc). Nevertheless, normalized reporter activation (Nluc/Fluc) showed an increase in pathway activation of the surviving cell pool up to 10 µM geldanamycin (Fig. 3a, Nluc/Fluc) where the general viability of the cells was only around 30%. We compared the results of the dual-luciferase approach to a metabolic assay (resazurin) and a dye exclusion assay (propidium iodide, PI) using flow cytometry. Using two unspecific toxins (guanidinium and Tween20) to induce cell death without activating HSF1, we measured Fluc, Nluc, resazurin, and PI staining. For guanidinium (Fig. 3b), the results of the different assays were very similar, when the cells started to die (PI), both Fluc and Nluc decreased just as the metabolic activity. For Tween20 (Fig. 3c), however, there were dramatic differences observable between protein content (Fluc and Nluc), living cells, and metabolic activity. While the protein content and PI values again fit well together (Fluc 22.4% and PI positive cells 21.8% at 1000 µM Tween20), the metabolic activity increased drastically (160% compared to untreated control cells) up to a Tween20 concentration of 320 µM even though the cells already died (PI). At a concentration of 1000 µM where PI values and Fluc values were comparable, the metabolic activity indicated a surplus viability (130% compared to 22% for PI and Fluc, *p* = 0.000013 with Student’s *t* test). At the highest concentration of 3200 µM Tween20, there was still more than 50% resazurin assay activity detectable, although there were hardly any viable cells left. A very similar picture was seen when the cells were additionally treated with geldanamycin to activate HSF1 (Fig. 3d). Consequently, an internal reference on the basis of a second luciferase allows a highly reliable detection of the cell viability and might substitute for a dye exclusion assay, when metabolic activity is not accurate enough as a mean of a viability assessment. However, for standard inducer experiments and long incubation times, the resazurin assay was in good agreement with the dual-luciferase measurements, representing an adequate and practical method for determining cell viability.

### Quantitative Comparison of HSF1 Activators

In order to generate an assay to directly and quantitatively compare the potency of different HSR inducers on HSF1 activation, we created a stable HSE reporter cell line and established a universal induction scheme. The X9-12H cell line was used to test 26 substances reported to activate the HSR. The range of tested concentrations shown in Table 1 was adapted from data found in the literature or determined by preliminary experiments. In Table 1, the lowest concentration leading to a significant increase in HSE reporter activity is shown as EC2 (effective concentration 2 indicating a twofold increase above untreated control cells). Further, the maximum induction (Imax) is given as fold increase over control cells together with the concentration leading to Imax (*C*^Imax^). For viability measurements of this large panel of experiments, we used the resazurin assay, whereas dual-luciferase measurements were applied for selected experiments (indicated in Table 1). Viability levels are shown for EC2, *C*^Imax^, and the highest concentration tested (max. conc.). 15 out of the 26 substances (or treatments) could be identified as HSF1 activators in our system. A large group of the substances that failed to elicit a response on the HSE reporter (BGP-15, arimoclomol, acetyl salicylic acid, mefenamic acid, iboprufen, and geranylgeranyl acetone) were described previously to not activate a HSR by themselves, but rather to act as co-inducers in combination with other stressors. These substances were also tested for their ability to co-activate HSF1 and the HSR (see below). The treatments resulting in the highest Imax were heat treatment (> 9000-fold) and the heavy metal cadmium (> 1000-fold). The most potent HSF1 activators (lowest EC2) were the Hsp90 inhibitors geldanamycin and SNX-2112 inducing the reporter at concentrations below 1 µM. To correlate activation of HSF1 on the artificial promoter in HEK293 cells to the endogenous HSR target Hsp72, we also conducted Western blot experiments in 2 distinct cell lines (HeLa and WI38) for selected inducers (TPCK, carbenoxolone, EtOH, MetOH, and pH) and found good accordance with our HSF1 reporter data (Fig. S4). In at least one of the two cell lines, Hsp72 protein induction could be seen already after 6 h except for MetOH where at a concentration of 3 M, the protein extract preparation failed.

**Table 1:** Comparison of HSF1 activators in the X9-12H cell line

Activator	Conc. tested (µM)	EC 2	Viability EC2	Imax	C ^Imax^	Viability Imax (%)	Viability max. conc. (%)	Selected references
Alcohols								
Ethanol	0.5 × 10^6^–3 × 10^6^	2 × 10^6^	38	289	2 × 10^6^	38	2	[48]
Methanol	0.5 × 10^6^–3 × 10^6^	3 × 10^6^	77	20.9	3 × 10^6^	77	77	[49]
Heavy metals								
CdSO_4_	1–100	1	110	1492	100	42	42	[50]
Hsp70 inhibitors								
VER155008	10–250	50	87	2.4	50	52	27	[51]
Hsp90 inhibitors								
SNX-2112 (dual luc)	0.01–25	0.25	95	13.7	25	58	58	[52]
Geldanamycin	0.01–25	0.05	88	14.6	25	16	16	[32]
Hydroxylamin derivatives								
BGP-15^#^	3–100	–	–	1.05	30	92	95	[53]
Arimoclomol^#^	0.25–50	–	–	1.0	0.5	83	89	[37]
NSAIDs								
Sulindac^#^	100–1000	1000	49	16.4	1000	49	6	[54]
Acetyl salicylic acid^#^	1000–300,000	–	–	1.26	1000	98	0	[54]
Mefenamic acid^#^	3 200–3,200,000	–	–	1.09	3200	105	22	[54]
Iboprufen^#^	100–1000	–	–	0.87	250	212	182	[54]
Osmotic stressors								
NaCl	10,000–100,000	–	–	1.0	25,000	120	89	[55]
Sorbit	10,000––300,000	–	–	1.0	30,000	119	29	[55]
Other drugs								
Carbenoxolone	100–1000	500	65	7.4	1000	40	40	[56]
Celastrol	5000–100,000	–	–	1.1	50,000	100	116	[57]
Resveratrol	50–500	–	––	0.7	50	91	38	[58]
Geranylgeranyl acetone^#^	10–300	–	–	1.21	100	113	50	[59]
Oxidative stressors								
H_2_O_2_	5000–25,000	1000	49	170.5	2500	29	1	[60]
Physical stress								
pH (MES)*	pH 7.4–4	pH 5	100	27.9	pH 5	100	6	[61]
pH (TRIS)*	pH 8–11	pH 10	124	5.6	pH 11	123	123	[61]
HS	40–47 °C	41 °C	100	9473	47,°C	33	33	[40]
Proteasome inhinitors								
MG132	3.2–100	10	100	51.1	100	64	64	[62]
Serine protease inhibitors								
TPCK	10–100	50	74	99.9	100	64	64	[63]
Unspecific stressors								
Guanidinium	5000–50,000	10,000	99	2	10,000	99	41	[42]
Tween20 (dual luc)	100–1000	–	–	0.9	100	123	168	[64]

X9-12H cells were treated with different potential inducers, and HSF1 activation is given as Nluc signal relative to untreated control cells. Viability was determined with a resazurin assay. Asterisks (*) indicate a 1 h treatment followed by 24 h recovery, and heat shock (HS) was performed for 10 min. All other substances were used for 24 h incubation. Dual-luciferase measurements for cell viability are indicated (dual luc), for all other treatments, a resazurin assay was used. All values represent the mean of at least two independent experiments

*EC2* effective concentration 2, *Imax* maximal induction, *C*^*Imax*^ concentration for maximal induction, *Viability Imax* viability seen at the concentration with maximal induction relative to untreated control cells, *viability max conc*. viability seen at the highest concentration tested relative to untreated control cells, potential co-inducers are indicated with ^#^

### Testing of Co-Inducers

**Fig. 4:**
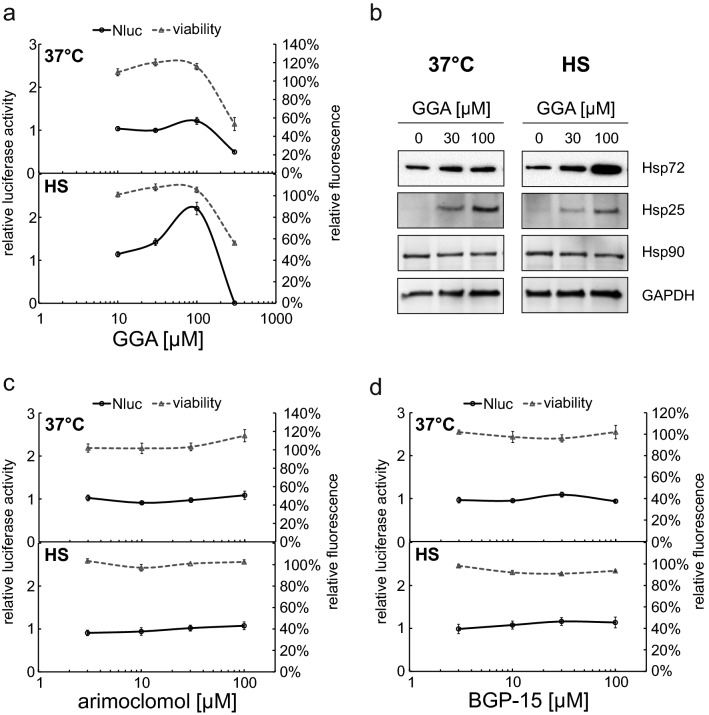
Analysis of potential HSR co-inducers. X9-12H cells were treated with different concentrations of geranylgeranylacetone (GGA, **a**), arimoclomol (**c**), and BGP-15 (**d**) for 1 h before a 1 h heat treatment at 42 °C was performed (HS) followed by 24 h recovery at 37 °C or a continuous cultivation at 37 °C (37 °C) for 24 h before luciferase and fluorescence measurement (**a**, **c,** and **d**). Left *Y*-axes (**a**, **c** and **d**) show relative luciferase activity as Nluc signal compared to cells without co-inducer. Right *X*-axes (**a**, **c** and **d**) show viability as relative fluorescence of resazurin compared to cells without co-inducer. All values are means of at least three independent experiments, error bars indicate SEM, Student’s *t* test was applied to estimate significance (see text). For Western blot analysis (**b**), WI38 cells were used and treated with GGA under identical conditions as in (**a**). Primary antibodies targeting Hsp72, Hsp25, Hsp90, and GAPDH were used

HSR co-inducers are characterized by their ability to additionally upregulate the expression of molecular chaperones in cells in the presence of different stress effectors or pathologic conditions such as heat shock, diabetes or ischemia [65]. To test the HSR enhancing ability on the level of HSF1 activation of some known co-inducers, we pre-treated the cells for 1 h with the drug followed by a 1 h heat treatment. We again analyzed luciferase activity (Fig. 4a, c and d) to detect direct effects on HSF1 and Hsp expression to capture the endogenous HSR (Fig. 4b) after 24 h of treatment. For actual co-inducers, we expected to see higher induction levels when combining inducer and heat treatment compared to heat treatment alone, although the co-inducer alone does not necessarily activate HSF1. In our hands, only geranylgeranylaceton (GGA), a known ulcer drug [66], displayed clear co-inducer abilities and the reporter as well as on Hsp72 protein level. Cells treated with 100 µM GGA alone displayed a slight increase in luciferase levels (1.22-fold). However, if the cells were heat treated in the presence of GGA, we observed a significant increase of luciferase levels (2.20-fold; *p* = 0.00009 with Student’s t test; Fig. 4a), while the viability of the cells was unaffected. Western blotting for Hsp72 confirmed the good co-inducer ability of GGA (Fig. 4b). Protein levels increased with heat treatment and were further raised by pre-treatment with 30 and 100 µM GGA. In contrast, this effect was not seen on the other Hsps analyzed (Hsp27 and Hsp90). Hsp27 was strongly induced by GGA already at 30 µM, but the expression was not increased by further heat treatment. Hsp90, on the other hand, was not induced at any condition. The two hydroxylamine (HA) derivatives, arimoclomol and BGP-15, were not able to upregulate luciferase levels in combination with heat treatment (Fig. 4c and d). We also tested the non-steroidal anti-inflammatory drugs (NSAIDs) acetyl salicylic acid (Fig. S5a), sulindac (Fig. S5b), mefenamic acid (Fig. S5c), and ibuprofen (Fig. S5d). Treatment with 0.32 mM sulindac resulted in a weak activation (1.43-fold). Additional heat treatment (42 °C) led to significant increase of these values (2.34-fold; *p* = 0.0038 with Student’s t test); however, at 1 mM (16-fold raise in luciferase levels), an additional heat treatment resulted in a dramatic drop of viability (Fig. S5b), indicating increased stress rather than a co-inducer effect for the additional heat treatment. None of the other NSAIDs were able to induce luciferase expression with or without heat treatment (Fig. S5). As a side observation, we saw that mefenamic acid (Fig. S5c) and ibuprofen (Fig. S5d) increased the fluorescence levels of the resazurin measurement similar to the effect of Tween20 (Fig. 3c and d), making them candidates to use the Fluc measurement instead of the metabolic activity for accurate viability assessment.

### HSF1 Activators for Potential Therapeutic Application

**Fig. 5:**
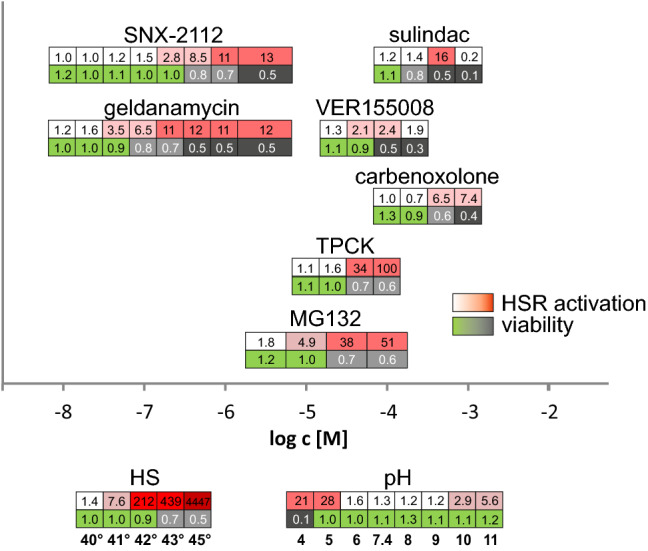
Quantitative comparison of potential therapeutic HSF1 activators. X9-12H cells were treated with different concentrations of the HSF1 activators for 24 h, heat treated for 10 min and analyzed after 24 h (HS) or treated with DMEM with different pH values for 1 h and then analyzed after 24 h recovery in DMEM pH 7.4 (pH). After treatment or recovery, viability was determined with a resazurin assay and Nluc was measured. Upper row indicates fold reporter activation compared to untreated control cells. Color code shows no induction in white to high induction in dark red. Lower row shows relative viability compared to untreated control cells with green (viable) to dark gray (survival below 50%). All values show the mean of at least 3 independent experiments with 8 replicates each. *X*-axis shows concentration in [M], temperature of heat treatment or pH of induction medium (Color figure online)

In order to be of therapeutic interest, an HSF1 activator should be able to specifically target the transcription factor without influencing other functions of the cell negatively. An easy way to assess general cell functions is the observation of metabolic activity as a viability marker. We, therefore, compared the dose response of the Nluc HSE reporter (Fig. 5, upper row) of potential therapeutic drugs to their viability data (Fig. 5, lower row). For comparison, we also show the response to heat treatment and changes in pH. Of the 26 drugs tested, we considered 7 of potential interest for therapeutic applications. Out of these, only the Hsp90 inhibitors (geldanamycin and SNX-2112), the Hsp70 inhibitor (VER155008), and the proteasome inhibitor MG132 were able to activate HSF1 at concentrations where the cell viability of the HEK293 cell line was not yet compromised. The highest activation of the HSE reporter, combined with uncompromised viability, showed the treatments with heat (42 °C) and low pH (pH 5) (212- and 28-fold reporter activation, respectively). Sulindac, carbenoxolone, and TPCK only activated HSF1 in combination with substantially reduced cell viability. Geldanamycin and SNX-2112 are highly potent activators of HSF1 and represent the only two drugs that would supposedly be able to reach pharmacologically relevant concentrations within body tissue.

## Discussion

Over the last decades, reports of potential HSF1 activators accumulated in the literature. They can either be used to study the molecular mechanisms of the HSR in detail, or manipulate HSF1 activity as a potential therapeutic approach in various diseases, ranging from neurodegenerative (Alzheimer’s, Parkinson’s disease and ALS) to metabolic diseases. Although reviewed summaries exist, a comparison of the potency of the different substances and treatments found in the literature is difficult because the effects were demonstrated in various cell lines or even in animals and with different assays [11, 25, 67, 68]. We focused on the detection of active HSF1 with an artificial HSE reporter cell line to exclude HSF-independent stress responses. The use of stable cell lines with luciferase as reporter gene allows a quantitative comparison of the different treatments and further facilitates upscaling of the experiments with the use of 96- or even 384-well plates. A factor that can strongly influence the result, is the choice of the cell line. We used HEK293 cells, because they are easy to grow, are of non-cancer origin, and are robust when treated with the drugs at high concentrations. To back up this approach and to correlate mere HSF1 activation to actual endogenous target gene expression, we included two further cell lines in our study to monitor Hsp expression: the human cervical carcinoma cell line HeLa and the human fibroblast cell line WI38. HeLa cells were shown to be strictly dependent on the expression of HSF1 [69] and are also commonly used for the analysis of the HSR in the literature. The fibroblasts originate from a healthy donor and are, therefore, a good model for studying pathway activity in a close to in vivo situation. The use of three different cell types generates a more general perspective of the obtained data although direct quantitative comparison was only performed in the HEK293-based reporter cell line.

In general, we could reproduce the HSF1-activating potential of most drugs; however, they vastly differed in their potency. Unspecific stress can impair the homeostasis of the cells and, thus, indirectly induce proteotoxic stress, which subsequently activates the HSR and HSF1. However, such unspecific stress affects the viability of the cells and, thus, is not compatible with an application in pathological conditions. A pharmacologic activation of HSF1 would ideally not generate any proteotoxic stress at all. The HSP90 inhibitors geldanamycin and SNX-2112 with detectable activation starting in the range of 100 nM seem to be most closely to this ideal condition. They show a small therapeutic window between the first activation of the HSF1 and the first effects on cell viability (Fig. 5). Compared to this, the Hsp70 inhibitor VER 155008 and the proteasome inhibitor MG132 show potencies more than 100-fold lower but still exhibit a window of activation with little effect on the viability. In principle, however, all these inhibitors do not directly activate HSF1, but instead block vital pathways of the cell thought to be essential for the elimination of proteotoxic stress conditions. Therapeutic application of these drugs, therefore, always will be a compromise between a loss and a gain of individual stress responses.

All other drugs seem to affect the HSF1 indirectly, with considerable decrease of the viability already before HSF1 activity can be detected. Interestingly, two very simple ways of HSF1 activation exhibited strong induction combined with little effects on viability. Both low and high pH values and, in particular heat, which appeared to be by far the strongest activator in our assays. Controlled changes in the pH might, therefore, be considered for therapeutic applications. Thus, acid treatment of wounds [70] could in part depend on a beneficial activation of the HSF1 in the affected cells. Taking into account the low effect on viability and the extraordinary levels of activation, heat might represent a direct activator of HSF1. Since heat often can destroy cells within short-time periods, it is possible that evolutionary pressure drove the cells to sensitively detect slight changes due to rising temperatures, in order to respond immediately with activation of emergency pathways. In any case, heat can easily be applied and controlled from the outside [71] and in our experiments combined strongest activation of HSF1 with the mildest effects on cell viability.

Celastrol, arimoclomol, and BGP-15 did not show any effect on HSF1 activation and endogenous target gene expression in our system although there are promising results from cell culture and animal models and even clinical trials for various diseases caused by dysregulated protein homeostasis (e.g., neurodegenerative diseases, lysosomal dysfunctions, and muscular dystrophy) [72–77]. The mechanism of Hsp upregulation for these substances is still poorly understood but thought to be related to a prolonged or eased HSE-HSF1 interaction [37, 78]. The fact that our systems failed to detect an increased HSR after application of these co-inducers, which were shown to work dependent on the presence of HSF1 [37, 78, 79], further leads to the interesting question of necessary co-factors or post-translational modifications of HSF1 not present in all cell lines. Geranylgeranyl acetone, on the other hand, was able to generate a co-inducing effect on the HSR, on the level of HSF1 activation in accordance with data found in the literature [80, 81]. Although the different reactions of the tested Hsps indicate that a non-HSF1-related effect might also be responsible for part of the observed co-inducer effect on protein level. Similarly, we observed a lack of co-inducing potential for NSAIDs. For most NSAIDs, it was suggested that they induce HSF1-HSE binding, but without transcriptional activation [54, 82, 83]. As co-inducers, they were demonstrated to promote HSF1 target gene expression when the cells were exposed to low-temperature heat treatment [82, 84]. In some cell types (mast cells, monocytes), they are able to induce Hsp72 expression without further treatment [85, 86]. We saw no effect on HSF1 activation in our system with or without additional application of a mild heat treatment except for sulindac, which was able to lead to a reporter activation even without heat treatment.

The aim of our work was to provide a quantitative comparison of the HSF1 activation potential of common HSR inducers. This should help choose candidates from the broad spectrum of inducers when studying HSF1 activity. The comparison with the literature data also corroborates the view that some inducers act via general mechanisms present in all different types of cells, whereas others are cell type specific and raise the possibilities to study different HSF1 regulations. It cannot be excluded that other HSFs participate in this regulation and indeed, reports exist that under certain conditions, HSF2 can exceed the activity of HSF1 [87]; however, the optimized binding site of the reporter in combination with the high transcriptional power of HSF1 should prefer the activity of this transcription factor.

The use of a general induction scheme also is a unique feature of this work, although it might in some cases lead to contradicting results. Nevertheless, the use of the very stable Nluc reporter protein should minimize the chance to miss HSF1 activation even after a long induction period. To evaluate the suitability of drug candidates for a therapeutic approach, the use of a reporter cell line in combination with a viability assay can only be the first step, because other parameters such as systemic toxicity, bioavailability, and metabolism have to be considered. These limitations led to dismissal of very promising HSR inducers from clinical trials [32]. However, if a potential drug fails to activate the reporter cell line with low cytotoxicity and at low concentrations, it can be excluded at an early stage of drug development. Further, the generated cell line cannot only be used to look for novel HSF1 activators, but also for HSF1 repressors as potential therapeutics for various cancer types [88].

## Supplementary Information

Below is the link to the electronic supplementary material.

Supplementary file1 (PDF 48 kb)

Supplementary file2 (PDF 83 kb)

Supplementary file3 (PDF 1357 kb)

Supplementary file4 (PDF 1595 kb)

Supplementary file5 (PDF 1371 kb)
