# Supplementary material for: Quantitative Comparison of HSF1 Activators
Source: Mol Biotechnol. 2022 Feb 26;64(8):873–87. doi: 10.1007/s12033-022-00467-3 (PMC9259536; doi:10.1007/s12033-022-00467-3)
Supplement: Supplementary file 1 — Supplementary file1 (PDF 48 kb) [file 12033_2022_467_MOESM1_ESM.pdf]

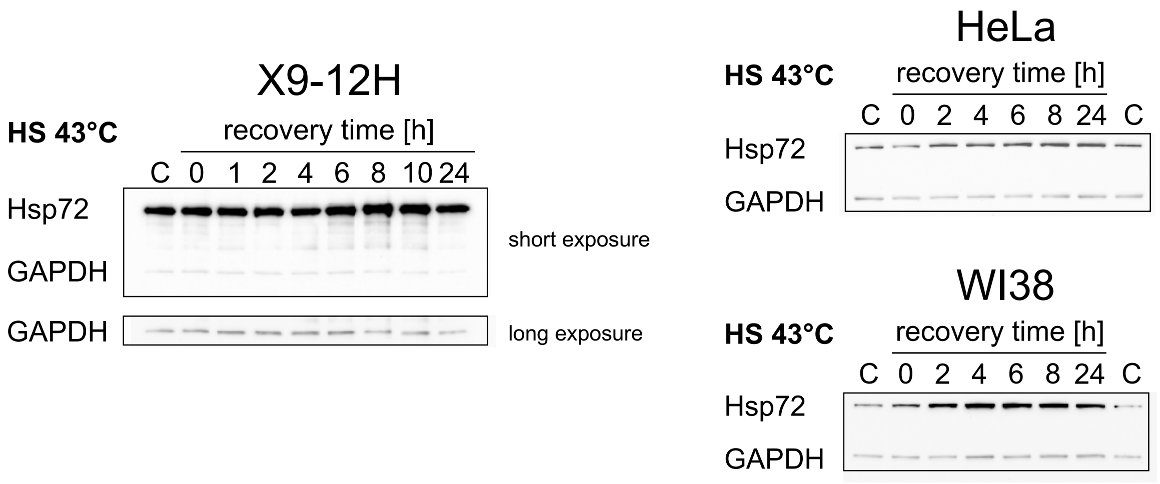

Fig. S1 Hsp72 protein expression after heat treatment. X9-12H, HeLa and WI38 cells were heat treated for 10 min at 43°C and whole cell protein extracts were taken at indicated time points. Control cells (C) were kept at 37°C. Western blot was performed with primary antibodies against Hsp72 and GAPDH
