# Supplementary material for: Quantitative Comparison of HSF1 Activators
Source: Mol Biotechnol. 2022 Feb 26;64(8):873–87. doi: 10.1007/s12033-022-00467-3 (PMC9259536; doi:10.1007/s12033-022-00467-3)
Supplement: Supplementary file 2 — Supplementary file2 (PDF 83 kb) [file 12033_2022_467_MOESM2_ESM.pdf]

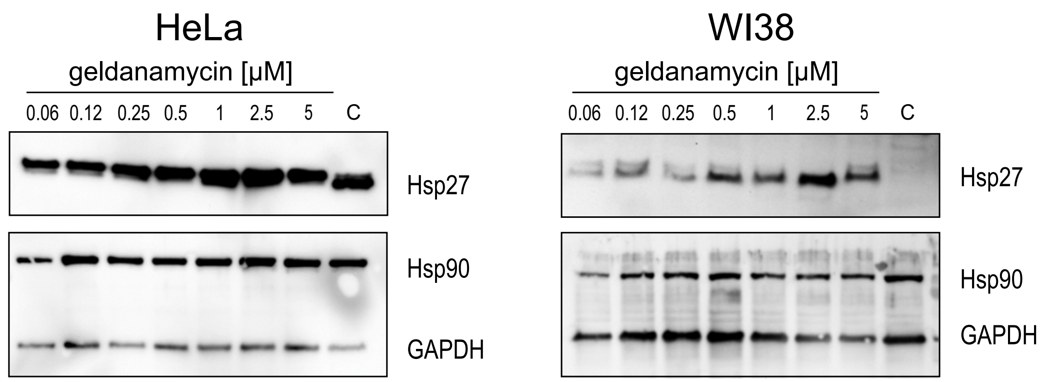

Fig. S2 Hsp27 and Hsp90 protein expression after geldanamycin treatment. HeLa and WI38 cells were incubated with different concentrations of geldanamycin and whole cell protein extracts were taken after 24 h. Control cells were untreated. Western blot was performed with primary antibodies against Hsp27, Hsp90 and GAPDH.
