# Supplementary material for: Quantitative Comparison of HSF1 Activators
Source: Mol Biotechnol. 2022 Feb 26;64(8):873–87. doi: 10.1007/s12033-022-00467-3 (PMC9259536; doi:10.1007/s12033-022-00467-3)
Supplement: Supplementary file 3 — Supplementary file3 (PDF 1357 kb) [file 12033_2022_467_MOESM3_ESM.pdf]

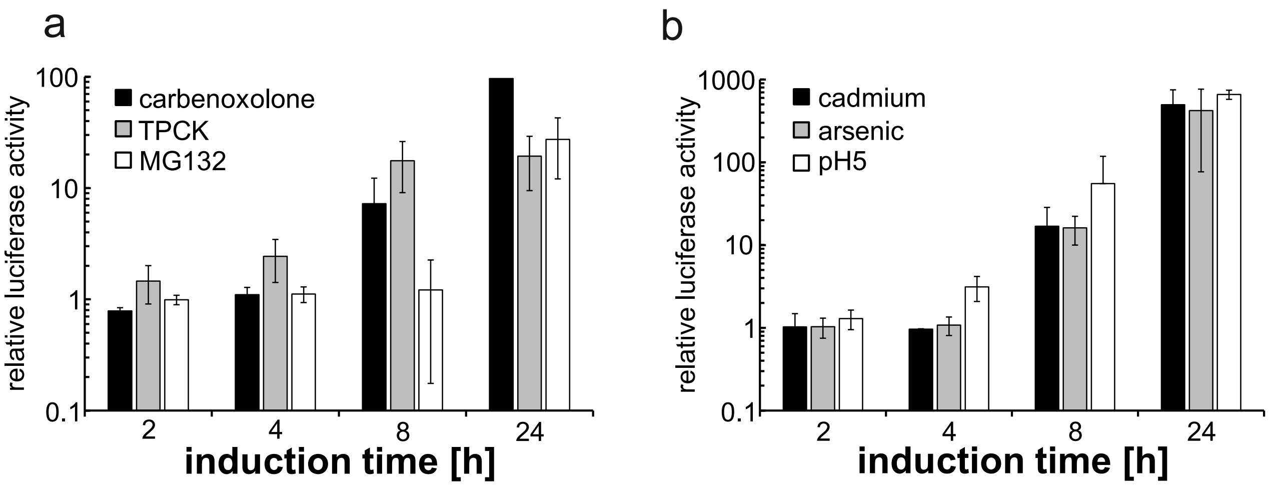

Fig. S3 Kinetics of the X9-12H reporter cell line after treatment with different HSR inducers. X9-12H cells were treated with carbenoxolone (500 µM), TPCK (50 µM), MG132 (10 µM) (a) and cadmium (CdSO4, 10 µM), arsenic (AsNaO2, 50 µM) (b) for indicated time points before luciferase measurement. For MES buffered DMEM pH 5 (b) cells were exposed for 1 h then washed and incubated for indicated time in fresh DMEM pH 7.4 before luciferase measurement. Y-axes show relative luciferase activity as Nluc signal compared to untreated control cells. All values show the means of at least two independent experiments, error bars indicate standard deviation (SD)
