# Supplementary material for: Quantitative Comparison of HSF1 Activators
Source: Mol Biotechnol. 2022 Feb 26;64(8):873–87. doi: 10.1007/s12033-022-00467-3 (PMC9259536; doi:10.1007/s12033-022-00467-3)
Supplement: Supplementary file 4 — Supplementary file4 (PDF 1595 kb) [file 12033_2022_467_MOESM4_ESM.pdf]

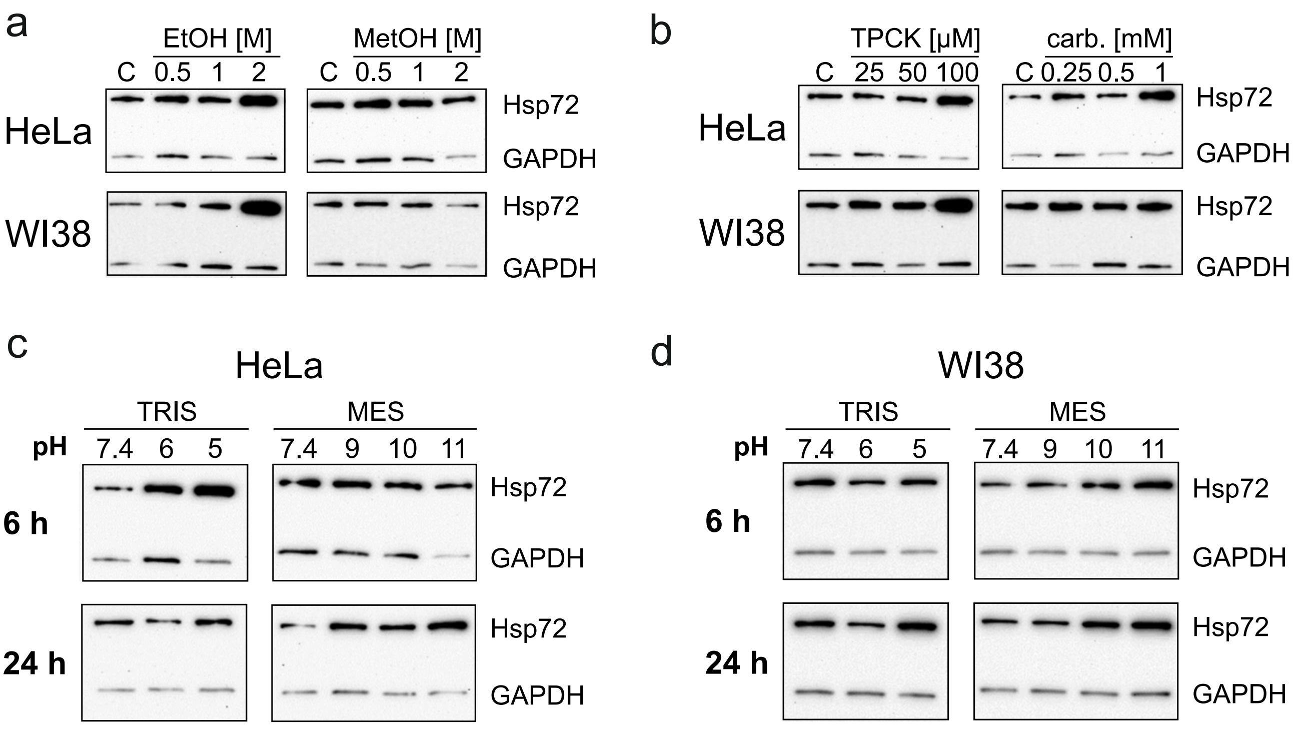

Fig. S4 Hsp72 protein expression after treatment with different inducers. HeLa and WI38 cells were treated with different concentrations of EtOH and MetOH for 1 h and then recovered in fresh DMEM for 6 h (a) or treated with different concentrations of TPCK and carbenoxolone (carb.) for 6 h (b) or DMEM buffered with TRIS or MES at different pH values for 1 h and afterwards recovered in fresh DMEM for 6 h or 24 h (c and d). After induction or recovery whole cell protein extracts were taken and analysed by Western blot with primary antibodies targeting Hsp72 and GAPDH
