# Supplementary material for: Quantitative Comparison of HSF1 Activators
Source: Mol Biotechnol. 2022 Feb 26;64(8):873–87. doi: 10.1007/s12033-022-00467-3 (PMC9259536; doi:10.1007/s12033-022-00467-3)
Supplement: Supplementary file 5 — Supplementary file5 (PDF 1371 kb) [file 12033_2022_467_MOESM5_ESM.pdf]

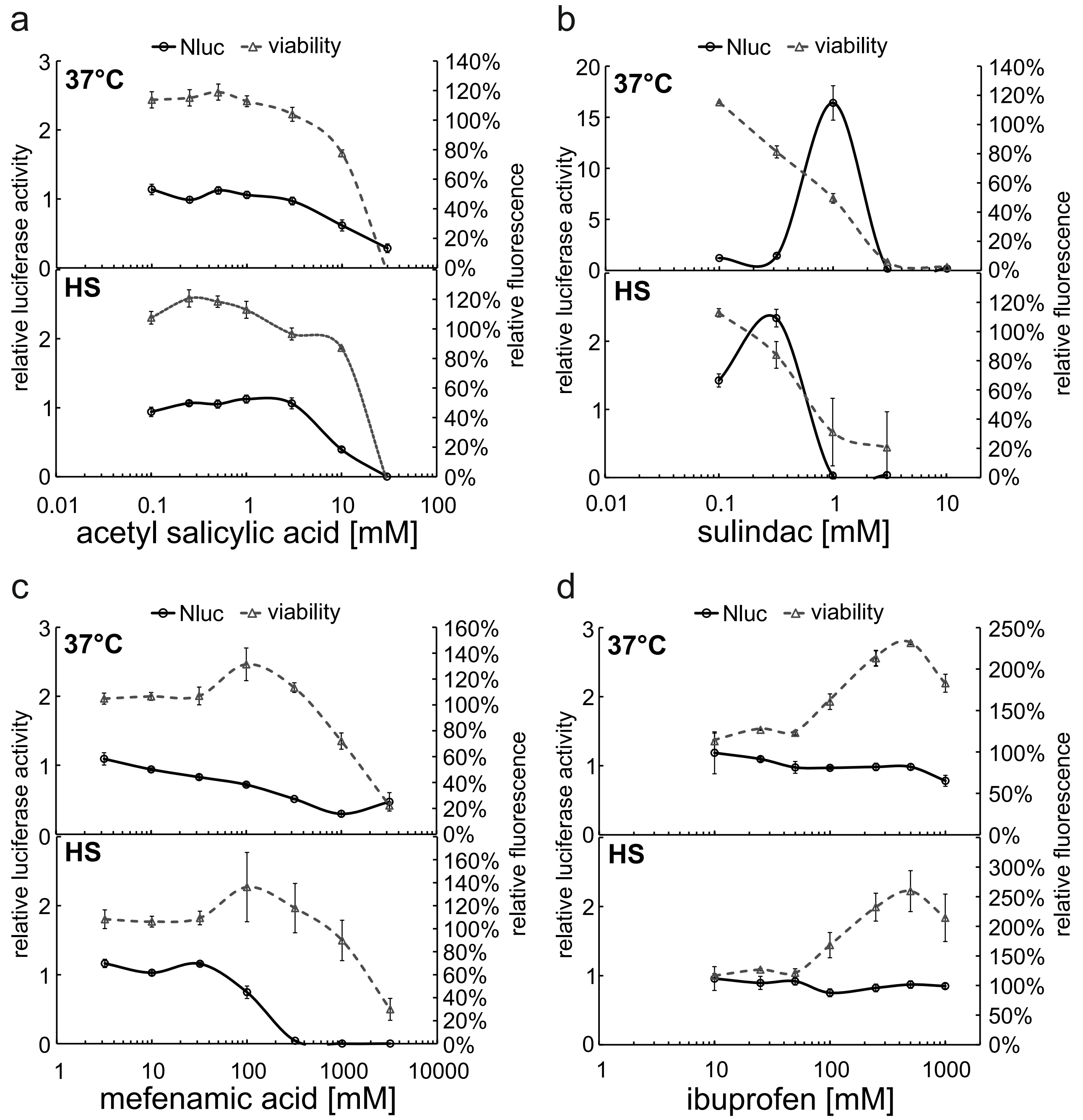

Fig. S5 NSAIDs as potential HSR inducers. X9-12H cells were treated with different concentrations of acetyl salicylic acid (a), sulindac (b), mefenamic acid (c) and ibuprofen (d) for 1 h before a 1 h heat shocked at 42°C was performed (HS) followed by 24 h recovery at 37°C or a continuous cultivation at 37°C (37°C) for 24 h before luciferase and fluorescence measurement. Left y-axes show relative luciferase activity as Nluc signal compared to cells without NSAID. Right x-axes show viability as relative fluorescence of resazurin compared to cells without NSAID. All values are means of at least three independent experiments, error bars indicate SEM
